# Toward personalized management of metastatic PPGL: decoding the implications of the ^177^Lu-DOTATATE Phase II interim analysis

**DOI:** 10.1097/JS9.0000000000005046

**Published:** 2026-03-17

**Authors:** Tonghu Liu, Junpeng Ji, Zechen Yan

**Affiliations:** a Department of Urology, Peking University Third Hospital, Beijing, China; b Department of Urology, The Third Affiliated Hospital, College of Clinical Medicine of Henan University of Science and Technology, Luoyang, China; c Department of Urology, The First Affiliated Hospital of Zhengzhou University, Zhengzhou, China; d Institute of Molecular Cancer Surgery, Zhengzhou University, Henan Province Engineering Research Center, Zhengzhou, Henan, China

*Dear Editor,*

We read with great interest the interim analysis by Lin *et al* on the Phase II trial of ^177^Lu-DOTATATE for progressive metastatic pheochromocytomas and paragangliomas (PPGL)[1]. This prospective, genetically stratified study conducted at the National Cancer Institute, provides critical high-level evidence for the treatment of PPGL, especially following the withdrawal of high-specific-activity ^131^I-MIBG in early 2024. The results confirm the clinical efficacy of ^177^Lu-DOTATATE particularly highlights the significant outcome differences between patients with SDHx mutations and those with sporadic disease, thereby offering valuable insights for personalized treatment strategies. We commend the authors for this important work and would like to offer the following perspectives to further clarify and extend its implications. This commentary adheres to the TITAN Guidelines 2025, which provide standards for declaring and utilizing artificial intelligence in scientific manuscripts[2].

First, the prospective design and genetic stratification effectively address the heterogeneity commonly encountered in retrospective studies. The notably inferior progression-free survival in the SDHx-mutated cohort (12.9 months vs 24.3 months) is a key finding. It underscores the importance of genotype in future trial design and therapeutic strategy development for PPGL. We recommend that subsequent research further investigate the differences between specific SDHB mutations and other SDHx variants and develop more robust risk-prediction models to help improve patient prognosis[3].

Second, the 17.8% incidence of grade 3 or higher catecholamine release syndrome (CRS) represents a unique and critical safety signal in PPGL therapy. Patients experiencing CRS may present with life-threatening symptoms such as extreme hypertension and ventricular arrhythmias[4]. The proactive management protocol proposed by the authors (α/β-blockade, ICU monitoring for high-risk patients) may benefit these patients to some extent. However, to optimize risk mitigation broadly, we suggest that future studies systematically report pretreatment catecholamine levels, the adequacy of blockade, and the precise temporal relationship between infusion and CRS onset. This will facilitate the establishment of a more accurate CRS risk-prediction model and standardized risk management pathways, ensuring the safe delivery of this effective therapy across diverse clinical settings.

Third, interestingly, the study observed sustained tumor shrinkage in most patients months after treatment, suggesting that an appropriate “watch-and-wait” strategy may be adopted before clear clinical deterioration or radiological progression occurs. However, the mechanism underlying this phenomenon remains unclear – is it due to a sustained radiation effect, immune activation, or both? Correlation analyses of blood biomarkers (e.g., chromogranin A) and immune profiling of posttreatment biopsy specimens in future studies may help elucidate this phenomenon, potentially revealing opportunities for synergistic combinations with immunotherapy.

Finally, with the recent approval of belzutifan, the sequencing of therapies for metastatic PPGL has become an urgent clinical challenge[5]. The efficacy and toxicity profiles of ^177^Lu-DOTATATE differ from those of tyrosine kinase inhibitors and HIF-2α antagonists. We recommend that future research compare these treatment modalities in greater detail regarding their efficacy, safety, and impact on quality of life. Large-scale randomized controlled trials are needed to determine the optimal treatment sequence or combination strategies for PPGL, particularly for high-risk SDHx-mutated tumors.

In summary, this study confirms the efficacy and safety of ^177^Lu-DOTATATE in treating progressive metastatic PPGL. Its prospective genetically stratified design provides a key foundation for personalized treatment. The key issues highlighted, such as genotype-based efficacy differences, CRS management, the mechanism of delayed response, and treatment sequencing, are all central to current clinical practice in PPGL. We look forward to future research further advancing the field toward more precise, safe, and orderly therapeutic approaches.

## Data Availability

Not applicable.
